# Detecting coevolution without phylogenetic trees? Tree-ignorant metrics of coevolution perform as well as tree-aware metrics

**DOI:** 10.1186/1471-2148-8-327

**Published:** 2008-12-03

**Authors:** J Gregory Caporaso, Sandra Smit, Brett C Easton, Lawrence Hunter, Gavin A Huttley, Rob Knight

**Affiliations:** 1 Department of Biochemistry and Molecular Genetics, University of Colorado Denver, Aurora, CO, USA; 2 Centre for Integrative Bioinformatics VU (IBIVU), Vrije Universiteit Amsterdam, 1081 HV Amsterdam, The Netherlands; 3 Computational Genomics Laboratory, John Curtin School of Medical Research, The Australian National University, Canberra, ACT, Australia; 4 Center for Computational Pharmacology, University of Colorado Denver, Aurora, CO, USA; 5 Department of Chemistry and Biochemistry, University of Colorado at Boulder, Boulder, CO, USA

## Abstract

**Background:**

Identifying coevolving positions in protein sequences has myriad applications, ranging from understanding and predicting the structure of single molecules to generating proteome-wide predictions of interactions. Algorithms for detecting coevolving positions can be classified into two categories: tree-aware, which incorporate knowledge of phylogeny, and tree-ignorant, which do not. Tree-ignorant methods are frequently orders of magnitude faster, but are widely held to be insufficiently accurate because of a confounding of shared ancestry with coevolution. We conjectured that by using a null distribution that appropriately controls for the shared-ancestry signal, tree-ignorant methods would exhibit equivalent statistical power to tree-aware methods. Using a novel t-test transformation of coevolution metrics, we systematically compared four tree-aware and five tree-ignorant coevolution algorithms, applying them to myoglobin and myosin. We further considered the influence of sequence recoding using reduced-state amino acid alphabets, a common tactic employed in coevolutionary analyses to improve both statistical and computational performance.

**Results:**

Consistent with our conjecture, the transformed tree-ignorant metrics (particularly Mutual Information) often outperformed the tree-aware metrics. Our examination of the effect of recoding suggested that charge-based alphabets were generally superior for identifying the stabilizing interactions in alpha helices. Performance was not always improved by recoding however, indicating that the choice of alphabet is critical.

**Conclusion:**

The results suggest that t-test transformation of tree-ignorant metrics can be sufficient to control for patterns arising from shared ancestry.

## Background

Knowledge of positions that coevolve in biological sequences can be applied to predict structures of RNAs [1-3] and proteins [4-7]; to predict intermolecular interactions [6,8]; to identify functionally important regions of molecules [9,10]; and to identify energetic pathways through molecules [11,12]. Coevolutionary analyses have frequently been performed on one or a few protein families. However, just as The Adaptive Evolution Database [13] allows proteome-wide studies of evolutionary rates, proteome-wide studies of coevolution could also be could performed if sufficiently fast and well-characterized methods for detecting coevolution were available.

As biological sequences are the product of an evolutionary process, it intuitively makes sense that the accuracy of analyses of the historical processes affecting them will be improved by explicit representation of those historical processes. Incorporation of phylogenetic information has benefited diverse classes of bioinformatics algorithms, including multiple sequence alignment [14], comparison of microbial communities [15], and functional annotation of genes [16]. Accordingly, incorporation of phylogenetic knowledge to control for patterns in biological sequence data that arise from ancestry is regarded as essential for coevolutionary analyses and best achieved by directly incorporating the phylogeny in the metric [7,17,18].

Many coevolution algorithms ('tree-aware' methods) have explicitly attempted to control for phylogeny (e.g., [4-6,9,17,19,20]), while others ('tree-ignorant' methods) have implicitly assumed a star phylogeny (e.g., [10,12,21,22]). Drawbacks have been identified for both approaches. Likelihood based tree-aware methods have the disadvantage of being sensitive to model mis-specification, a property common to all likelihood methods, and generally have a much longer compute time than tree-ignorant methods. Tree-ignorant methods are thought to have decreased specificity due to confounding of correlations arising from selective pressure with correlations arising from shared ancestry represented by the phylogeny [9,23,24]. Past evaluations of the effect of tree topology on the performance of coevolution algorithms have used simulated data, and have confirmed that non-star tree topologies can cause false positives [21,23,25,26].

Clearly, controlling for shared ancestry is essential but approaches that do so without explicitly representing the phylogenetic tree are possible. We hypothesized a tree-ignorant statistic can be informative if it is compared to a distribution of the same statistic with the same embedded ancestry but variable in coevolution. In this case, the shared ancestry origin of correlated evolution dominates the background distribution. A greater magnitude of correlated evolution than this background is thus evidence of coevolution.

An additional consideration for estimating coevolution is that encoding protein alignments with reduced-state amino acid alphabets reduces computational complexity, and may also increase statistical power [5,7,21,27]. In a reduced-state alphabet, the twenty amino acids are collapsed to a smaller number of states. For example, a three-state 'charge' alphabet can be achieved by treating His, Lys, and Arg as the 'positively charged' state; Asp and Glu as the 'negatively charged' state; and, all other residues as the 'uncharged' state. The recoding chosen for a group of sequences constitutes an explicit hypothesis concerning the primary biochemical property subjected to coevolutionary pressures by natural selection. The motivation for choosing to recode sequences stems from the bias-variance trade-off, where statistical models with fewer parameters have lower variance (and typically greater statistical power) but more bias. A coevolution algorithm applied to sequences with fewer states should therefore have more power to identify pairs of positions which coevolve as a result of the physicochemical property being modeled (e.g., charge) because variability within each state is hidden. Information is lost in recoding to a reduced-state alphabet, so the power for detecting coevolution arising from other properties of amino acid residues (e.g., side-chain volume) decreases. The sensitivity of inference concerning coevolution to the encoding choice is unclear.

Evaluations of coevolution algorithms on simulated data elucidate the strengths and limitations of the algorithms, but are forced to rely on simplifying assumptions about the biological systems being modeled. Evaluations on biological data are therefore important for understanding how an algorithm will perform under more realistic circumstances. Biologically relevant evaluations are difficult however, because we have little knowledge of when sequence positions truly coevolve, and therefore do not have a good idea of what the true positives are.

Different approaches have been employed to define coevolutionary positive-controls. Individual cases of coevolution are directly supported by observation of variants known to cause disease in one species in another species [28]. This class of variant has been reported for both RNA and protein coding genes and does exhibit strong statistical evidence for coevolution [27]. The suitability of this class of variation for examining the properties of coevolution, however, is low for both practical and biological reasons: the number of cases for which there is sufficient data from related species is low; and, the identification of these variants as pathological suggests the selection coefficients operating on them is very strong and thus may not be representative of the strength of selection responsible for most coevolution.

An alternative approach has been to focus on candidate molecular-structure influences likely to be subjected to natural selection. Past evaluations on (non-simulated) RNA alignments have treated base pairs as positive controls and all other pairs as negative controls [1,2,20,24]. These have been useful for comparing algorithms on RNA, but it is not clear that performance on RNA alignments translates well to performance on protein alignments because interactions between residues in proteins are generally more complex. Protein gold standards have been designed to evaluate a method's ability to identify residue contacts in tertiary structure by defining residues pairs within a certain *C*_*β *_distance in a representative crystal structure as positive controls, and all other pairs as negative controls [5,9,20-22,29-32]. The set of residue pairs within a small *C*_*β *_distance in tertiary structure in a representative crystal structure is recognized as a coarse criteria because it is not clear that close physical proximity is an essential precondition for coevolution [32], and because a single crystal structure may not accurately describe the tertiary contacts in all sequences in the alignment.

To complement residue-contact-based comparisons, we present a novel secondary-structure-based method for comparing coevolution methods where the known periodic stabilizing interactions between stacked residues in protein alpha helices are taken as positive controls. Double-mutant studies of protein alpha helices have shown that stacked residues in alpha helices interact to stabilize the helix [33-36]. Statistical analyses support these results by showing that the interactions are present in diverse families of alpha helices [5,9,37-39]. Stabilization is thought to result from ionic interaction, aromatic-aromatic interaction, or hydrogen bonding between stacked side chains. Although there has been discussion on the validity of these studies [40], biophysical and statistical analyses continue to support the case for stabilizing interactions. These interactions occur between the stacked positions in the alpha helix, or the positions separated by three residues in primary structure (*i*, *i *+ 4) (where *i *refers to the sequence position), and to a lesser extent between positions separated by two residues (*i*, *i *+ 3), corresponding to the 3.6 residue per turn periodicity of the alpha helix. Since interactions between stacked residues appear important for alpha helix stability, we argue that positions should coevolve to conserve these interactions. Methods for detecting coevolution in proteins should therefore identify stacked residues in alpha helices, as illustrated in [5,26,38,41] providing a positive control for coevolution detection algorithms. We emphasize that we are not presenting the coevolution algorithms that we test as methods for detecting alpha helices from sequence data, but rather exploiting the known regular structure of the helix as a gold standard for detecting coevolution: methods for detecting coevolution should, at minimum, be able to recapture these regularities.

We report an assessment of the hypothesis that appropriately transformed tree-ignorant metrics have similar statistical power to tree-aware approaches by performing a systematic comparison of nine coevolution algorithms. Five of the algorithms – Mutual Information (MI), Normalized Mutual Information (NMI) [21], Resampled Mutual Information (RMI) (introduced here), Statistical Coupling Analysis (SCA) [12], and Corrected Mutual Information (MIp) [22] – use multiple sequence alignments but no phylogenetic trees. The other four methods – LnLCorr [5,7], Ancestral States (AS) [4,17], the Generalized Continuous-Time Markov Process Coevolutionary Algorithm (GCTMPCA) [3,6], and CoMap [20,24] – use multiple sequence alignments and phylogenetic trees. We additionally considered including the method described in [9], but opted to include NMI and MIp instead since they are expected to perform better than the former (K. Wollenberg, personal communication). The algorithms were compared by application to real (i.e., non-simulated) protein sequence alignments.

In a secondary study, our alignments are recoded in 52 different reduced-state amino acid alphabets to evaluate the utility of amino acid alphabets which model different chemical properties, and to test the hypothesis that alphabets with fewer states are generally better for detecting coevolution.

## Results

Five tree-ignorant methods and four tree-aware methods for detecting coevolving positions were compared on four multiple sequence alignments using the full amino acid alphabet and, when applicable, 52 reduced amino acid alphabets. Two of the alignments, tetrapod myoglobin (42 sequences, 153 positions) and chordate myosin rod (114 sequences, 1064 positions), represent mostly alpha helical protein sequences and thus our positive controls. The other two alignments are matched negative controls generated by shuffling the order of positions in each observed alignment to remove all structural information.

**Figure 1:**
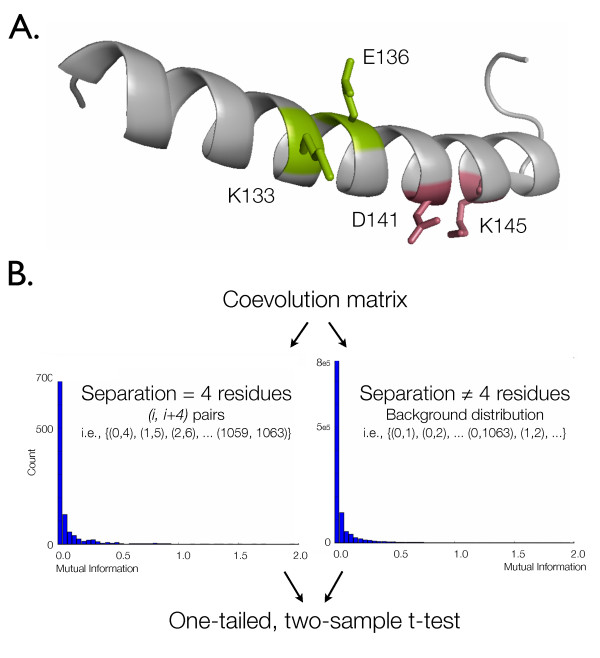
**Diagram of alpha-helix-based evaluation**. (A) Stacked positions in alpha helices have been observed to coevolve to maintain ionic interactions which stabilize the helix. The periodicity of the alpha helix is 3.6 residues per turn, so stacked positions are the (*i*, *i *+ 3) and (*i*, *i *+ 4) position pairs, where *i *is the position number. K133/E136 form an (*i*, *i *+ 3) pair capable of an ionic interaction; D141/K145 are an (*i*, *i *+ 4) pair capable of an ionic interaction. The structure is a single helix extracted from 1 MBD, a sperm whale myoglobin structure, diagramed with PyMol. (B) The distribution of coevolution scores from (*i*, *i *+ *n*) position pairs are compared against a background distribution generated from all other position pairs in the same alignment using a one-tailed, two-sample t-test. The scale of the y-axes differ due to the large difference in the number of position pairs in each distribution. The p-values resulting from these tests are those presented in this document. These t-test p-values were compared against p-values generated with other statistical tests to confirm the applicability of the t-test to these data (see Methods).

The statistical significance of coevolution metrics between alignment columns separated by a specified distance was determined using the t-test transformation (Figure 1). Applying a coevolution algorithm to an alignment results in a 'coevolution matrix,' where each position in the lower triangle contains a pairwise coevolutionary score. Each coevolution matrix, constructed based on a combination of method, alignment, and alphabet, was evaluated to determine to what degree the combination allowed detection of the periodicity of the alpha helix. The distribution of scores from (*i*, *i *+ *n*) positions in a coevolution matrix were compared with the distribution of all other scores in the same matrix using a two-sample t-test. Significant p-values at *n *= 3 or *n *= 4 were taken as evidence of a method's ability to detect coevolution, as these are the stacked positions in alpha helices which are expected to coevolve to retain alpha helix stability. Although the coevolutionary scores are not directly comparable between the methods, the t-test transformation standardizes the metrics, allowing evaluation of the relative performance of each method. We note here that although the distance matrix structure of the result matrices violates the independence clause of the t-test, we validated the robustness of results using a non-parametric matrix permutation test (described in Methods).

### Tree-aware versus tree-ignorant methods

The tree-ignorant methods are more capable of detecting coevolutionary signal at (*i*, *i *+ 3) and (*i*, *i *+ 4) in myoglobin than the tree-aware methods (Table 1A, row 1, p = 1.50 × 10^-3 ^and row 3, p = 4.97 × 10^-13^, respectively). There is no statistically significant difference in performance between the tree-aware methods on myoglobin or the myoglobin negative control (Table 2). Between the tree-ignorant methods, there is a statistically significant performance difference at (*i*, *i *+ 3) in favor of MIp, NMI, and SCA (Table 3A, row 1); and at (*i*, *i *+ 4) in favor of NMI and SCA (Table 3A, row 2). MI incurred more Type 1 error than the other methods (Table 3B, row 1, p = 2.71 × 10^-3^).

**Table 1:** Comparison of tree-aware methods and tree-ignorant methods.

(A) Tetrapod Myoglobin						
	Tree-aware		Tree-ignorant			
	*p *≤ *α*	*p *> *α*	*p *≤ *α*	*p *> *α*	*χ*^2^	*p *– *value*
**(i,i+3) w best SCA (0.9)**	3	125	**32**	**233**	**10.08**	**1.50 **× **10^-3^**

(i,i+3) w worst SCA (0.5)	3	125	23	242	5.61	1.79 × 10^-2^

**(i,i+4) w best SCA (0.8)**	14	114	**128**	**137**	**52.22**	**4.97 **× **10^-13^**

**(i,i+4) w worst SCA (0.4)**	14	114	**100**	**165**	**30.10**	**4.10 **× **10^-8^**

(B) Randomized Tetrapod Myoglobin						
	Tree-aware		Tree-ignorant			
	*p *≤ *α*	*p *> *α*	*p *≤ *α*	*p *> *α*	*χ*^2^	*p *– *value*

(i,i+3) w best SCA (0.9)	1	127	4	261	0.36	5.46 × 10^-1^

(i,i+3) w worst SCA (0.5)	1	127	9	256	2.38	1.23 × 10^-1^

(i,i+4) w best SCA (0.8)	0	128	1	264	0.48	4.87 × 10^-1^

(i,i+4) w worst SCA (0.4)	0	128	1	264	0.48	4.87 × 10^-1^

(C) Chordate Myosin						
	Tree-aware		Tree-ignorant			
	*p *≤ *α*	*p *> *α*	*p *≤ *α*	*p *> *α*	*χ*^2^	*p *– *value*

(i,i+3) w best SCA (0.4)	54	14	221	44	0.60	4.40 × 10^-1^

(i,i+3) w worst SCA (0.9)	54	14	198	67	0.65	4.21 × 10^-1^

(i,i+4) w best SCA (0.6)	58	10	209	56	1.41	2.36 × 10^-1^

**(i,i+4) w worst SCA (0.9)**	**58**	**10**	177	88	**8.92**	**2.82 **× **10^-3^**

(D) Randomized Chordate Myosin						
	Tree-aware		Tree-ignorant			
	*p *≤ *α*	*p *> *α*	*p *≤ *α*	*p *> *α*	*χ*^2^	*p *– *value*

(i,i+3) w best SCA (0.4)	7	61	56	209	4.14	4.18 × 10^-2^

(i,i+3) w worst SCA (0.9)	7	61	55	210	3.91	4.81 × 10^-2^

(i,i+4) w best SCA (0.6)	0	68	0	265	n/a	n/a

(i,i+4) w worst SCA (0.9)	0	68	0	265	n/a	n/a

*χ*^2 ^goodness-of-fit tests comparing all applicable tree-aware and all tree-ignorant methods for detecting alpha helix periodicity at separations of (*i*, *i *+ 3) and (*i*, *i *+ 4), using all reduced-state amino acid alphabets, where applicable. Some of the methods (LnLCorr07 and GCTMPCA) were not run on myosin (see text), causing different numbers of runs on the myosin versus the myoglobin data sets. Additionally, the reduced-state alphabets were not applicable to all tree-aware methods, causing the sum of the p-value counts to differ for the tree-aware versus tree-ignorant methods. Bolded rows highlight statistically significant *χ*^2 ^results, indicating a difference between the tree-aware and tree-ignorant populations. Bold counts in these rows highlight which class of methods achieved the higher ratio of significant to insignificant scores. Counts are calculated including both the empirically determined best and worst SCA cutoffs to illustrate the effect of this free parameter on method performance, and with the empirically validated optimal *ε *= 0.70 value for GCTMPCA. The data suggest that tree-ignorant methods perform better on the myoglobin alignment (A), and there is no statistically significant difference in the classes on the myosin rod domain alignment except when using the empirically determined worst SCA cutoff choice, when the tree-aware methods perform better (C). The myoglobin (B) and myosin (D) negative controls suggest that there is no significant difference in the Type 1 error incurred by the classes of methods. *α *= 0.01.

**Table 2:** Tree-aware methods compared on myoglobin and myosin.

(A) Tetrapod Myoglobin												
	GCTMPCA (*ε *= 0.70)		LnLCorr99		LnLCorr07		Ancestral States		CoMap			
	*p *≤ *α*	*p *> *α*	*p *≤ *α*	*p *> *α*	*p *≤ *α*	*p *> *α*	*p *≤ *α*	*p *> *α*	*p *≤ *α*	*p *> *α*	*χ*^2^	*p *– *value*
(i,i+3)	0	53	1	5	0	7	2	51	0	9	7.51	1.11 × 10^-1^

(i,i+4)	10	43	0	6	0	7	4	49	0	9	6.75	1.50 × 10^-1^

(B) Randomized Tetrapod Myoglobin												
	GCTMPCA (*ε *= 0.70)		LnLCorr99		LnLCorr07		Ancestral States		CoMap			
	*p *≤ *α*	*p *> *α*	*p *≤ *α*	*p *> *α*	*p *≤ *α*	*p *> *α*	*p *≤ *α*	*p *> *α*	*p *≤ *α*	*p *> *α*	*χ*^2^	*p *– *value*

(i,i+3)	1	52	0	6	0	7	0	53	0	9	1.43	8.40 × 10^-1^

(i,i+4)	0	53	0	6	0	7	0	53	0	9	n/a	n/a

(C) Chordate Myosin												
	GCTMPCA (*ε *= 0.70)		LnLCorr99		LnLCorr07		Ancestral States		CoMap			
	*p *≤ *α*	*p *> *α*	*p *≤ *α*	*p *> *α*	*p *≤ *α*	*p *> *α*	*p *≤ *α*	*p *> *α*	*p *≤ *α*	*p *> *α*	*χ*^2^	*p *– *value*

(i,i+3)	-	-	5	1	-	-	41	12	8	1	0.69	7.09 × 10^-1^

**(i,i+4)**	-	-	2	4	-	-	**48**	**5**	8	1	**14.18**	**8.33 × 10^-3^**

(D) Randomized Chordate Myosin												
	GCTMPCA (*ε *= 0.70)		LnLCorr99		LnLCorr07		Ancestral States		CoMap			
	*p *≤ *α*	*p *> *α*	*p *≤ *α*	*p *> *α*	*p *≤ *α*	*p *> *α*	*p *≤ *α*	*p *> *α*	*p *≤ *α*	*p *> *α*	*χ*^2^	*p *– *value*

(i,i+3)	-	-	0	6	-	-	7	46	0	9	2.21	3.31 × 10^-1^

(i,i+4)	-	-	0	6	-	-	0	53	0	9	n/a	n/a

*χ*^2 ^goodness-of-fit tests comparing the performance of tree-aware methods for detecting alpha helix periodicity at (*i*, *i *+ 3) and (*i*, *i *+ 4). Counts are calculated for GCTMPCA with the empirically validated optimal *ε *= 0.70. In most cases, there is no statistically significant difference in performance between the tree-aware methods with the single exception being (*i*, *i*+ 4) in myosin, where AS and CoMap out-perform LnLCorr99. Data is not presented on the myosin alignment for tree-aware methods which proved too computationally intensive to be practical. Counts in Table 2 can be directly compared with counts in Table 3. *α *= 0.01.

**Table 3:** Tree-ignorant methods compared on myoglobin and myosin.

(A) Tetrapod Myoglobin												
	MI		NMI		RMI		SCA		MIP			
	*p *≤ *α*	*p *> *α*	*p *≤ *α*	*p *> *α*	*p *≤ *α*	*p *> *α*	*p *≤ *α*	*p *> *α*	*p *≤ *α*	*p *> *α*	*χ*^2^	*p *– *value*
**(i,i+3)**	2	51	9	44	0	53	10	43	**11**	**42**	**17.98**	**1.24 **× **10^-3^**

**(i,i+4)**	32	21	**41**	**12**	1	52	36	17	18	35	**79.28**	**2.48 × 10^-16^**

(B) Randomized Tetrapod Myoglobin												
	MI		NMI		RMI		SCA		MIP			
	*p *≤ *α*	*p *> *α*	*p *≤ *α*	*p *> *α*	*p *≤ *α*	*p *> *α*	*p *≤ *α*	*p *> *α*	*p *≤ *α*	*p *> *α*	*χ*^2^	*p *– *value*

**(i,i+3)**	**4**	**49**	0	53	0	53	0	53	0	53	**16.25**	**2.71 × 10^-3^**

(i,i+4)	0	53	0	53	0	53	0	53	1	52	4.02	4.04 × 10^-1^

(C) Chordate Myosin												
	MI		NMI		RMI		SCA		MIP			
	*p *≤ *α*	*p *> *α*	*p *≤ *α*	*p *> *α*	*p *≤ *α*	*p *> *α*	*p *≤ *α*	*p *> *α*	*p *≤ *α*	*p *> *α*	*χ*^2^	*p *– *value*

**(i,i+3)**	**51**	**2**	46	7	38	15	40	13	46	7	**14.83**	**5.08 × 10^-3^**

**(i,i+4)**	**52**	**1**	44	9	26	27	44	9	43	10	**41.30**	**2.33 × 10^-8^**

(D) Randomized Chordate Myosin												
	MI		NMI		RMI		SCA		MIP			
	*p *≤ *α*	*p *> *α*	*p *≤ *α*	*p *> *α*	*p *≤ *α*	*p *> *α*	*p *≤ *α*	*p *> *α*	*p *≤ *α*	*p *> *α*	*χ*^2^	*p *– *value*

**(i,i+3)**	5	48	18	35	4	49	2	51	**27**	**26**	**53.30**	**7.38 × 10^-11^**

(i,i+4)	0	53	0	53	0	53	0	53	0	53	n/a	n/a

*χ*^2 ^goodness-of-fit tests comparing the performance of tree-ignorant methods for detecting alpha helix periodicity at (*i*, *i *+ 3) and (*i*, *i *+ 4). Bolded rows highlight statistically significant *χ*^2 ^results, indicating a difference between the methods. Bold counts in these rows highlight which method achieved the highest ratio of significant to insignificant scores. Counts are calculated using the empirically determined optimal SCA cutoff for each positive control data point. SCA cutoff is 0.9 for A-B row 1, 0.8 for A-B row 2, 0.4 for C-D row 1, and 0.6 for C-D row 2. *α *= 0.01.

**Figure 2:**
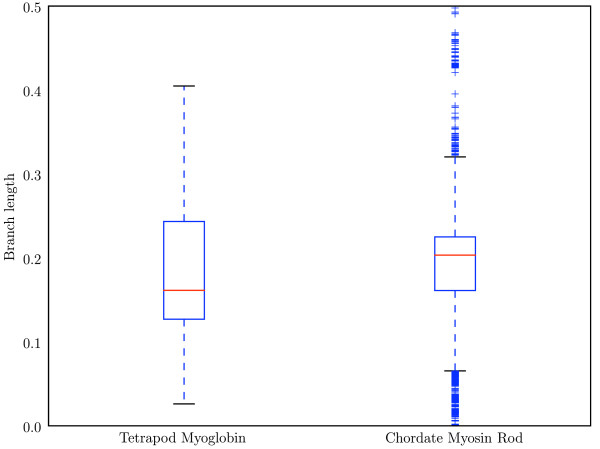
**Branch lengths associated with the myoglobin and myosin alignments**. Red line indicates the median value, and the top and bottom of the box indicate the upper and lower quartile values, respectively. Whiskers represent the largest and smallest values within 1.5 × IQR (inter-quartile range), and pluses represent outliers, or points outside of 1.5 × IQR. The distribution of branch lengths in the myosin tree is clearly wider and more variable than the distribution of branch lengths in the myoglobin tree.

In myosin, there is no significant difference in the performance of the tree-aware and tree-ignorant methods at (*i*, *i *+ 3) or (*i*, *i *+ 4) after SCA cutoff optimization, but before SCA optimization the tree-aware algorithms achieve better performance at (*i*, *i *+ 4) (Table 1C). The absence of a significant difference could reflect either real properties of the methods, or arise from reduced statistical power of this particular comparison. There is more variability in the branch lengths in the myosin data set (Figure 2), suggesting the possibility that as the relationships between sequences becomes more variable, accounting for those relationships becomes more important. However, the statistical power of the method comparisons are not identical between the myoglobin and myosin cases. Due to the computational intensity of GCTMPCA and LnLCorr07, these methods were not practical to run on the myosin alignment and tree. (Single runs of GCTMPCA and LnLCorr07 on the myosin rod were stopped after running for greater than 78 processor hours and 383 processor hours, respectively.) As a result, the myosin case has a reduced number of observations compared with that for myoglobin which will reduce the statistical power to identify differences between the method classes. Comparing LnLCorr99, CoMap, and AS on myosin shows that there is no statistically significant difference in these methods at (*i*, *i *+ 3) (Table 2C, row 1) but that AS and CoMap outperform LnLCorr99 at (*i*, *i *+ 4) (Table 2C, row 2, p = 8.33 × 10^-4^). Of the tree-ignorant methods, there was a statistically significant difference in the performance of the individual methods (Table 3C), and MI achieved the highest ratio of significant to insignificant scores, and fared well in terms of Type 1 error (Table 3D). On the myosin negative control, MIp achieved significantly more false positives than the other methods (Table 3D, row 1, p = 7.38 × 10^-11^). The ability of MI to out-perform all of the other methods, including the tree-aware methods, is extremely surprising.

**Figure 3:**
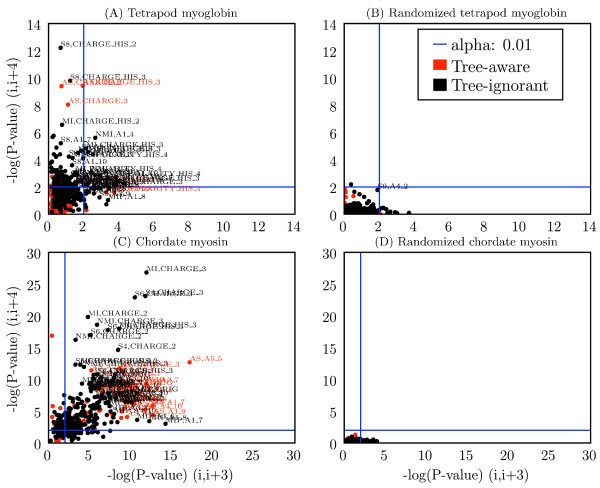
**Performance of method, alphabet combinations for detecting signal at (*i*, *i *+ 3) and (*i*, *i *+4)**. Each combination of method and alphabet is represented as a single point, with coordinates defined by the -log(p-value) at (*i*, *i *+ 3) (x-axis) and (*i*, *i *+ 4) (y-axis) for the (A) tetrapod myoglobin alignment, (B) randomized tetrapod myoglobin alignment, (C) chordate myosin rod alignment, and (D) randomized chordate myosin rod alignment. Blue lines indicate a significance threshold of *α *= 0.01. SCA cutoffs included for (A-B) are 0.9 and 0.8, and for (C-D) are 0.4 and 0.6. These represent the best cutoff values at (*i*, *i *+3) and (*i*, *i *+4), respectively, for each positive control data set. Label key: method, alphabet, so S8, CHARGE_HIS_2 refers to SCA with cutoff = 0.80, and the CHARGE_HIS_2 alphabet. Method abbreviations: AS: Ancestral States; L07: LnLCorr07; L99: LnLCorr99; MI: Mutual Information; NMI: Normalized Mutual Information; RMI: Resampled Mutual Information; S*n*: Statistical Coupling Analysis, cutoff = *n/*10; MIP, Corrected Mutual Information; G*n*: Generalized Continuous-Time Markov Process Coevolutionary Algorithm, *ε *= *n*/10; CM: CoMap. Alphabet definitions in Table 5.

Figure 3 shows the relative performance of all methods and parameter settings (alphabet choice, SCA cutoff and GCTMPCA *ε*). These data illustrate the results of the *χ*^2 ^tests (Figure 3A,C), and the negative controls confirm the validity of the evaluations by consistently showing no coevolution where it is not expected (Figure 3B,D).

**Figure 4:**
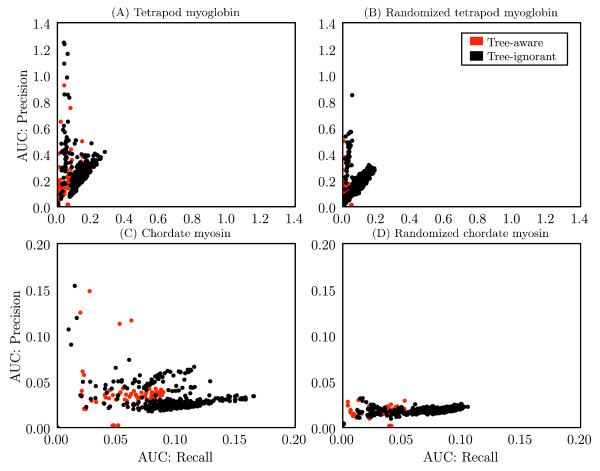
**Performance of method, alphabet combinations for detecting individual coevolving positions**. Each combination of method and alphabet is represented as a single point, with coordinates defined by area under the curve (AUC) values for recall (y-axis) and precision (x-axis) for the (A) tetrapod myoglobin alignment, (B) randomized tetrapod myoglobin alignment, (C) chordate myosin rod alignment, and (D) randomized chordate myosin rod alignment. The computation of AUC values is described in Methods.

**Figure 5:**
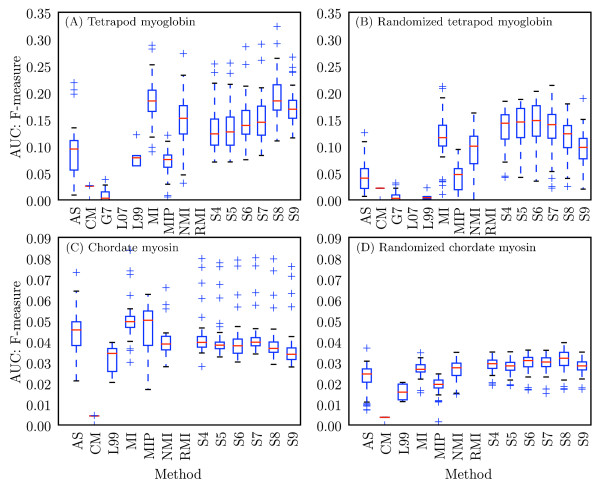
**Distribution of F-measures obtained with each method for all alphabets for detecting individual coevolving positions**. Modified box plots illustrate the distribution of F-measures obtained with a given method over all alphabets. Values are F-measure area under the curve (AUC) scores for (A) tetrapod myoglobin, (B) randomized tetrapod myoglobin, (C) chordate myosin rod, and (D) randomized chordate myosin rod. The computation of AUC values is described in Methods. Red lines indicate the median AUC, and the top and bottom of the boxes indicate the upper and lower quartile values, respectively. Whiskers represent the largest and smallest AUC values within 1.5 × IQR (inter-quartile range), and pluses represent outliers, or points outside of 1.5 × IQR. Methods with more condensed distributions are those that appear more robust to alphabet choice. AS: Ancestral States; L07: LnLCorr07; L99: LnLCorr99; MI: Mutual Information; NMI: Normalized Mutual Information; RMI: Resampled Mutual Information; S*n*: Statistical Coupling Analysis, cutoff = *n/*10; MIP, Corrected Mutual Information; G*n*: Generalized Continuous-Time Markov Process Coevolutionary Algorithm, *ε *= *n*/10; CM: CoMap.

The tree-aware and tree-ignorant methods were additionally evaluated on their ability to identify individual coevolving pairs by comparing area under the curve (AUC) scores for precision and recall (see Methods for definitions) for each method, alphabet combination (Figure 4). On the myoglobin data set, the tree-ignorant methods achieved higher AUC scores for precision and recall than the tree-aware methods. On the myosin alignment, the tree-ignorant methods achieved higher AUC scores for recall than the tree-aware methods, but the highest precision scores appear similar between the two classes of methods. On the myoglobin negative control, the tree-ignorant methods achieved higher AUC scores for precision and recall, suggesting more false positives for the tree-ignorant methods. On the myosin negative control, precision appears similar between the two classes, while the tree-ignorant methods appear to have achieved higher recall, indicating more false positives. Because the negative controls are a shuffled version of the original alignments, the total hits count is the same for the positive and negative control coevolution matrices for a given method, alphabet combination (with the exception of the LnLCorr methods, where the coevolution scores differ slightly between positive and negative control alignments). Due to the difficulty of defining positives and negatives (discussed in Methods), the precision, recall, and F-measure values for the negative controls are less meaningful than for the positive control, but are provided for completeness. Precision and recall results are summarized via F-measure in Figure 5, which presents the F-measure AUCs achieved by each method with each alphabet. The median F-measure AUCs appear higher for the tree-ignorant methods in both positive and negative controls. Precision, recall, F-measure, total hits, and AUC data for all method, alphabet combinations are provided [see Additional file 1].

### Alphabet reduction

To evaluate the effect of the size and type of reduced-state alphabets on algorithm performance, each alignment was recoded with 52 reduced-state alphabets. Coevolution algorithms were applied to each recoded alignment in addition to the original (full-alphabet) alignments. The 45 'Atchley-factor' alphabets are based on five metrics (A1–A5) presented in [42], and are used primarily to evaluate the effect of alphabet size. The 7 'rationally-designed' alphabets were developed based more canonical amino acid metrics, and are used primarily to evaluate the effect of alphabet type. Alphabet names describe the alphabet type (i.e., the property being modeled) followed by alphabet size (i.e., the number of states). For example, A1_4 refers to the A1-based alphabet with four states, and CHARGE_3 refers to the charge-based alphabet with three states. Alphabet definitions are presented in Table 5.

The data illustrate markedly different results with different alphabet definitions, both in terms of number of states and amino acid property modeled.

#### The effect of alphabet size

In the myoglobin alignment (42 sequences by 153 positions), there is no clear relationship between performance and number of alphabet states (Table 4A). The three of thirty-five correlations that are significant after correction for multiple comparisons are positive correlations, suggesting increased performance with more states. In the negative control (Table 4B) however, the ratio of significant to insignificant correlations is similar. These data therefore do not strongly support a relationship between alphabet size and method performance.

**Table 4:** Alphabet size and performance.

(A) Tetrapod Myoglobin										
Method	A1		A2		A3		A4		A5	
	*τ*	p-value	*τ*	p-value	*τ*	p-value	*τ*	p-value	*τ*	p-value
MI	-0.20	4.8 × 10^-1^	0.42	1.1 × 10^-1^	0.16	6.0 × 10^-1^	-0.33	2.2 × 10^-1^	0.24	3.8 × 10^-1^

NMI	-0.29	2.9 × 10^-1^	0.24	3.8 × 10^-1^	0.24	3.8 × 10^-1^	0.16	6.0 × 10^-1^	0.56	**2.9 **× **10^-3^**

RMI	0.11	7.3 × 10^-1^	0.33	2.2 × 10^-1^	-0.51	**4.7 **× **10^-3^**	-0.02	1.0	0.64	**9.1 **× **10^-3^**

SCA8	-0.24	3.8 × 10^-1^	0.78	**9.5 **× 10^-34^*	0.56	**2.9 **× **10^-3^**	-0.02	1.0	0.87	**1.2 **× 10^-34^*

MIp	0.16	6.0 × 10^-1^	0.47	7.3 × 10^-2^	0.29	2.9 × 10^-1^	-0.11	7.3 × 10^-1^	0.78	**9.5 **× 10^-34 ^*

AS	-0.02	1.0	0.64	**9.1 **× **10^-3^**	0.29	2.9 × 10^-1^	-0.47	7.3 × 10^-2^	0.07	8.6 × 10^-1^

G7	0.16	6.0 × 10^-1^	0.64	**9.1 **× **10^-3^**	0.33	2.2 × 10^-1^	0.42	1.1 × 10^-1^	0.47	7.3 × 10^-2^

(B) Randomized Tetrapod Myoglobin										
Method	A1		A2		A3		A4		A5	
	*τ*	p-value	*τ*	p-value	*τ*	p-value	*τ*	p-value	*τ*	p-value

MI	-0.38	1.6 × 10^-1^	-0.29	2.9 × 10^-1^	-0.07	8.6 × 10^-1^	0.16	6.0 × 10^-1^	0.60	**1.7 **× **10^-3^**

NMI	-0.29	2.9 × 10^-1^	-0.16	6.0 × 10^-1^	-0.47	7.3 × 10^-2^	-0.07	8.6 × 10^-1^	0.16	6.0 × 10^-1^

RMI	-0.38	1.6 × 10^-1^	-0.29	2.9 × 10^-1^	-0.11	7.3 × 10^-1^	-0.16	6.0 × 10^-1^	0.56	**2.9 **× **10^-3^**

SCA8	-0.73	**2 2 **× **10^-3^**	-0.69	**4.7 **× **10^-3^**	-0.60	**1.7 **× **10^-3^**	-0.64	**9.1 **× **10^-3^**	0.07	8.6 × 10^-1^

MIp	-0.38	1.6 × 10^-1^	-0.69	**4.7 **× **10^-3^**	-0.42	1.1 × 10^-1^	-0.11	7.3 × 10^-1^	0.29	2.9 × 10^-1^

AS	-0.56	**2.9 **× **10^-3^**	-0.42	1.1 × 10^-1^	-0.38	1.6 × 10^-1^	-0.38	1.6 × 10^-1^	-0.24	3.8 × 10^-1^

G7	0.60	**1.7 **× **10^-3^**	0.29	2.9 × 10^-1^	-0.29	2.9 × 10^-1^	0.56	**2.9 **× **10^-3^**	0.20	4.8 × 10^-1^

(C) Chordate Myosin										
Method	A1		A2		A3		A4		A5	
	*τ*	p-value	*τ*	p-value	*τ*	p-value	*τ*	p-value	*τ*	p-value

MI	0.78	**9.5 **× 10^-34^*	0.78	**9.5 **× 10^-34^*	0.64	**9.1 **× **10^-3^**	0.60	**1.7 **× **10^-3^**	0.82	**3.6 **× 10^-34^*

NMI	0.69	**4.7 **× **10^-3^**	0.78	**9.5 **× 10^-34^*	0.56	**2.9 **× **10^-3^**	0.29	2.9 × 10^-1^	0.82	**3.6 **× 10^-34^*

RMI	0.29	2.9 × 10^-1^	0.29	2.9 × 10^-1^	-0.07	8.6 × 10^-1^	-0.51	**4.7 **× **10^-3^**	0.60	**1.7 **× **10^-3^**

SCA6	0.56	**2.9 **× **10^-3^**	0.20	4.8 × 10^-1^	0.16	6.0 × 10^-1^	-0.07	8.6 × 10^-1^	0.16	6.0 × 10^-1^

MIp	0.78	**9.5 **× 10^-34^*	0.78	**9.5 **× 10^-34^*	0.69	**4**:**7 **× **10^-3^**	0.60	**1.7 **× **10^-3^**	0.73	**2.2 **× **10^-3^**

AS	-0.16	6.0 × 10^-1^	0.29	2.9 × 10^-1^	0.73	**2**:**2 **× **10^-3^**	0.42	1.1 × 10^-1^	0.29	2.9 × 10^-1^

(D) Randomized Chordate Myosin										
Method	A1		A2		A3		A4		A5	
	*τ*	p-value	*τ*	p-value	*τ*	p-value	*τ*	p-value	*τ*	p-value

MI	-0.51	**4.7 **× **10^-3^**	-0.33	2.2 × 10^-1^	-0.07	8.6 × 10^-1^	0.16	6.0 × 10^-1^	-0.16	6.0 × 10^-1^

NMI	-0.20	4.8 × 10^-1^	-0.78	**9.5 **× 10^-34^*	-0.29	2.9 × 10^-1^	0.51	**4.7 **× **10^-3^**	0.20	4.8 × 10^-1^

RMI	-0.33	2.2 × 10^-1^	-0.60	**1.7 **× **10^-3^**	-0.24	3.8 × 10^-1^	0.16	6.0 × 10^-1^	-0.11	7.3 × 10^-1^

SCA6	-0.16	6.0 × 10^-1^	-0.56	**2.9 **× **10^-3^**	-0.60	**1.7 **× **10^-3^**	0.20	4.8 × 10^-1^	-0.24	3.8 × 10^-1^

MIp	-0.29	2.9 × 10^-1^	-0.47	7.3 × 10^-2^	-0.64	**9.1 **× **10^-3^**	0.38	1.6 × 10^-1^	-0.51	**4.7 **× **10^-3^**

AS	0.20	4.8 × 10^-1^	0.20	4.8 × 10^-1^	-0.11	7.3 × 10^-1^	0.02	1.0	-0.11	7.3 × 10^-1^

For each of the five heuristically defined alphabets, Kendall correlation coefficients (*τ*) and corresponding p-values are provided to illustrate the relationship between performance rank and alphabet size. Bolded values highlight p-values = 0.05, and starred values indicated p-values significant after adjustment for multiple comparisons (A-B Bonferroni-adjusted *α *= 0.0014, C-D Bonferroni-adjusted *α *= 0.0017). Tau range: [-1., 1.]. A positive *τ *value indicates a positive correlation (i.e., increased alphabet size is associated with increased performance), and a negative *τ *value indicates a negative correlation (i.e., decreased alphabet size is associated with increased performance).

In the myosin rod alignment (114 sequences by 1064 positions) a positive correlation frequently exists between alphabet size and ability to detect alpha helix periodicity (Table 4C), particularly for MI, NMI, and MIp. This suggests that more alphabet states improve performance for these methods on the myosin rod. Seven of the thirty correlation coefficients are significant after correction for multiple comparisons, compared with one of thirty in the negative control (Table 4D).

#### The effect of alphabet type

**Figure 6:**
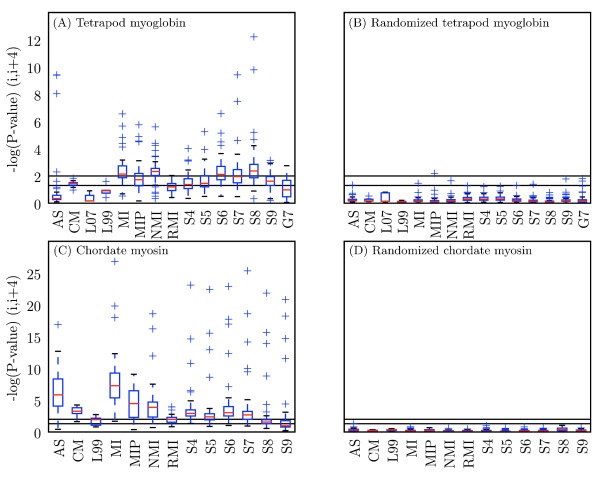
**Distribution of scores obtained with each method for all alphabets**. Modified box plots illustrate the distribution of scores obtained with a given method over all alphabets. Values are -log(p-value) at (*i*, *i *+ 4) for (A) tetrapod myoglobin, (B) randomized tetrapod myoglobin, (C) chordate myosin rod, and (D) randomized chordate myosin rod. Black lines indicate statistical significance threshold of *α *= 0.05 (bottom line) and *α *= 0.01 (top line). Red lines indicate the median value, and the top and bottom of the boxes indicate the upper and lower quartile values, respectively. Whiskers represent the largest and smallest values within 1.5 × IQR (inter-quartile range), and pluses represent outliers, or points outside of 1.5 × IQR. Methods with more condensed distributions are those that appear more robust to alphabet choice. AS: Ancestral States; L07: LnLCorr07; L99: LnLCorr99; MI: Mutual Information; NMI: Normalized Mutual Information; RMI: Resampled Mutual Information; S*n*: Statistical Coupling Analysis, cutoff = *n/*10; MIP, Corrected Mutual Information; G*n*: Generalized Continuous-Time Markov Process Coevolutionary Algorithm, *ε *= *n*/10; CM: CoMap.

The 'rationally-designed' alphabets generally out-perform the Atchley-factor alphabets [see Additional file 2], although the A1-based and A5-based alphabets were frequently among the top performing alphabets. The CHARGE_* alphabets very commonly yield the most significant p-values at (*i*, *i *+ 4) in both alignments. As A1 and A5 are charge and polarity factors, these were expected to perform well. In myoglobin, of the methods that identified (*i*, *i *+ 4) pairs with statistical significance at *α *= 0.01, seventeen of the thirty-five top-performing alphabets were the rationally designed alphabets. Eleven of the eighteen top-performing Atchley-factor alphabets were A1- or A5-based. In myosin, fifteen of the thirty top performing alphabets were rationally designed, and five of the fourteen top-performing Atchley-factor alphabets were A1- or A5-based. One of the top-performing alphabets for MIp was the unreduced alphabet (ORIG). It is not clear why the A3- and A4-based alphabets performed better in myosin, although these mostly did well with RMI. RMI exhibits very little variance in p-value based on choice of alphabet (Figure 6), which may make p-value-based ranking less meaningful. Because LnLCorr99 and CoMap use alphabets differently from the other algorithms (see Methods), the corresponding counts of Atchley-factor and 'rationally-designed' alphabets were not included in these computations. Top-performing alphabets for these methods are however presented [see Additional file 2].

The four-state alphabet based on Atchley Factor 4 (A1_4) shows up frequently in the top alphabets, and on inspection appears to mirror the decisions that might be made if manually defining an alphabet based on this metric (Bin 1: CVILF, Bin 2: MWAGS, Bin 3: TPYHQ, Bin 4: NDERK). These bins mimic natural break-points in this metric, with the exception of Q being in Bin 3. The generally high performance of coevolution methods working on alignments encoded in A1_4 suggests that it may compliment the use of the more canonical reduced-state alphabets, and that other Atchley-factor alphabets defined based on natural breaks in the data may also yield good results.

### GCTMPCA and SCA parameters

SCA and GCTMPCA both require a single parameter (cutoff and *ε*, respectively) from the user. Experimentation with the values of these parameters was incorporated into the analyses, and was found to have a strong effect on method performance. [3] and [6] suggest 0.70 as an optimal setting for *ε*. This was empirically validated here by comparing performance for detecting signal at (*i*, *i *+ 3) and (*i*, *i *+ 4) in myoglobin using the full amino acid alphabet and two reduced-state alphabets (data not shown).

The optimal value for the SCA cutoff parameter was empirically found to be variable (see distributions of performance by cutoff in Figure 6A,C) and did not match the values obtained as recommended in [12]. When applied to the alignments without alphabet reduction, the recommended steps identify 0.947 as the optimal value for myosin, and 1.0 as the optimal value for myoglobin (meaning there are not enough sequences for the analysis). The empirically-determined optimal cutoff for detecting coevolutionary signal at (*i*, *i *+ 4) in myosin was 0.6, and in myoglobin was 0.8, when no alphabet reduction was applied. These results suggest that the steps presented in [12] will not always identify the optimal cutoff, and experimentation with the cutoff value should therefore be incorporated into applications of SCA.

## Discussion

### Tree-aware versus tree-ignorant techniques

Tree-ignorant coevolution algorithms matched or out-performed the tree-aware coevolution algorithms for identifying coevolving positions in alpha helices. With the exception of CoMap, the tree-aware methods also generally required vastly more compute time than the tree-ignorant methods, despite the slowest of these algorithms (GCTMPCA and LnLCorr) being implemented in C while the other algorithms are predominantly implemented in Python (C implementations are frequently orders of magnitude faster than equivalent implementations in Python). The C++ implementation of CoMap was the fastest of the methods. In some cases, standard Mutual Information out-performed all other algorithms (Table 3C). These observations suggest that when transformed in a manner that adjusts for shared ancestry, as demonstrated here, tree-ignorant methods can be more reliable than tree-aware methods. Although consideration of additional genes with substantial sampled lineages may support a convergence in robustness between the two method classes, the computational performance advantage of tree-ignorant methods establishes them as the metric of choice for comprehensive surveys of molecular coevolution.

Figure 6 illustrates that with the correct parameter choices (i.e., amino acid alphabet and free parameters to algorithms) nearly all of the methods can identify the (*i*, *i *+ 4) stacked residues in the two alpha helical proteins. In most applications however, the 'correct' amino acid alphabet will not be known prior to the analysis, and it will not be practical to optimize the free parameters and alphabet. For example, if trying to infer protein-protein interactions on a genome-wide scale based on coevolutionary relationships, true positives would not be known. Even if a subset of known interactions could be used for optimization, the compute time would be prohibitive. Methods that are fast and robust to parameter choice are preferable. In myoglobin, MI and NMI stand out in this respect: the distribution of scores achieved by these two metrics is skewed toward statistical significance compared with the other methods (Figure 6A). The same appears true in myosin, with RMI additionally appearing extremely robust to alphabet choice but achieving a lower median p-value compared to MI and NMI (Figure 6C). AS, MIp, and CoMap generally perform well on myosin, although some alphabet choices with AS and MIp can lead to very poor performance. The variance introduced by the cutoff parameter choice with SCA is large in both alignments, and GCTMPCA and LnLCorr do not appear robust to alphabet choice and achieve median p-values below the *α *= 0.05 significance threshold, suggesting that these methods would be less useful in cases where the choice of alphabet cannot be optimized or confidently specified *a priori*.

The ability of tree-ignorant methods, and MI in particular, to out-perform tree-aware methods is a seemingly surprising result given previous demonstrations of high false positives for this method class. We have argued, and our results demonstrate, the t-test transformation provides sufficient control for the shared ancestry cause of associations. Because every column (position) in an alignment has the same underlying relationship among the rows (sequences), every estimated pairwise coevolution score will have this influence in common. Pairs with a true coevolutionary history will have both that shared ancestry and the additional influence of coevolution, and so should stand out from the non-coevolving pairs.

### Alphabet size

Larger alphabets frequently improve performance of coevolution algorithms (MI, NMI, and MIp in particular) on the myosin data set, but there is no obvious relationship between alphabet size and coevolution algorithm performance on the myoglobin data. The differences observed on myoglobin and myosin may be explainable by differences in the quantity of input data. The myosin rod data set has more sequences than the myoglobin data set, and therefore more states to observe when calculating a pairwise coevolution score between two columns. Grouping residues via reduced alphabets is expected to improve statistical performance by providing a clearer picture of the distribution of residue types. The additional observations (sequences) present in the myosin alignment appear sufficient to describe the residue distribution without recoding, and the information loss associated with recoding in fewer states may therefore result in decreased statistical performance. The lack of a significant positive or negative correlation between alphabet size and statistical performance on myoglobin suggests that (unlike on myosin) higher-state alphabets are not better, and that a generic benefit associated with fewer-state alphabets may only arise on alignments with less sequences than the myoglobin data.

An alternative explanation for the positive correlation between alphabet size and performance in myosin is that the Atchley-factor alphabets are not useful categorizations of the data. If true, as the number of states increases (and the alphabets become more similar to the full amino acid alphabet) they should perform better. The strongest correlations however are achieved with the A1 and A5 alphabets, which are the Atchley-factor alphabets that are expected to perform the best (because they model charge and polarity, the features known to be important to interactions in alpha helices). Some of the best performances overall were achieved using these alphabets [see Additional file 2], so this explanation appears unlikely.

Much previous work, including [5] which used the same myoglobin alignment and tree, has focused on the assumption that smaller alphabets are generally better. If true, a negative correlation should exist between alphabet size and method performance. This is not observed, suggesting that in practice smaller alphabets do not necessarily improve statistical performance.

### Alphabet type

If probing for coevolution resulting from a specific type of physicochemical interaction, a reduced alphabet which models that physicochemical property in fewer states should increase power. There is serious risk, however, of losing power by using the incorrect reduced alphabet. For example, the p-value for identifying signal at (*i*, *i *+ 4) with MI increased (i.e., became less significant) from 1.5 × 10^-27 ^with the CHARGE_3 alphabet, to 1.0 × 10^-3 ^with the SIZE_2 alphabet. For all methods analyzed on myosin with reduced alphabets – AS, MI, NMI, RMI, MIp, and SCA – the SIZE_2 alphabet always resulted in less significant p-values than the CHARGE_3 alphabet. This illustrates that recoding with the correct alphabet (CHARGE_3 here) will highlight a coevolutionary relationship based on that property, but that recoding with an incorrect alphabet (SIZE_2 here) will obscure the coevolutionary signal. Non-sensical alphabet recoding should destroy the coevolutionary signal. If the interaction type (e.g., charge, size) is not known ahead of time, using the unreduced alphabet can provide useful results and should be safer than making an uninformed choice of reduced alphabet.

The importance of choosing the 'correct' reduced alphabet is illustrated by variable performance of a single algorithm even when the alphabet choices have the same number of states. For detecting coevolutionary signal between stacked residues in alpha helices, the reduced alphabets which model charge/polarity perform the best. These include the four CHARGE_* alphabets (CHARGE_2, CHARGE_3, CHARGE_HIS_2, CHARGE_HIS_3) and the alphabets based on Atchley factors 1 and 5. These would not be the best alphabets if the interactions were based on a different physicochemical property.

The success of charge-based alphabets for identifying stacked positions in alpha helices confirms the previous biochemical and statistical observations, and suggests that alphabet recoding can be applied to probe interaction types. For example, if a coevolutionary interaction is apparent when working with a size-based alphabet, but disappears when working with a charge-based alphabet, the interaction is likely more related to size than charge. This is observed when looking at (*i*, *i *+ 1) pairs in the myosin rod using MI with the SIZE_2 and CHARGE_3 alphabets (data not shown). Coevolution on the basis of side-chain volume between (*i*, *i *+ 1) pairs, which form the set of residues which are presumably closest to one-another in the folded protein, seems likely. Classification of interaction types based on these characteristics is often likely to be overly simplistic, but probing interactions this way may serve as a starting point for more in-depth analysis. Care should be taken however to avoid spurious conclusions arising from multiple comparisons when repeatedly applying a coevolution algorithm to the same alignment recoded with multiple alphabets.

### Utility of alpha-helical protein alignments for comparing coevolution algorithms

This article presents a comparison of nine coevolution detection methods on two different molecules which are entirely (myosin rod) or almost entirely (myoglobin) alpha helical. The two alignments are different in terms of number of sequences, diversity of sequences, and sequence length, yet in both cases the algorithms are able to detect (to varying degrees) the periodicity of the alpha helix. The coevolution of stacked residues in protein alpha helices, identifiable computationally and supported by double-mutant studies, makes alignments of alpha helices useful for comparing techniques for detecting coevolution. Unfortunately, we cannot generalize this approach to beta sheets, because the length of the beta strands is highly variable and there is thus no consistent periodic signal expected to be consistent across different proteins.

**Figure 7:**
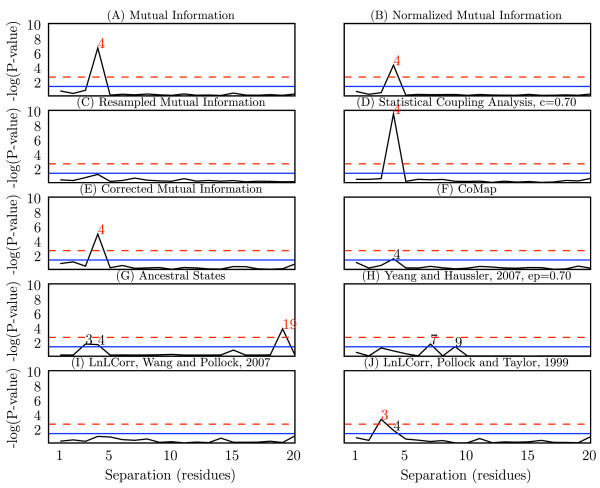
**Coevolutionary signal in myoglobin at (*i*, *i *+ *n*) pairs for n ranging from 1 to 20**. -log(p-values) are presented for each separation of *n *residues (*i*, *i *+ *n*) for *n *ranging from 1 to 20. Biochemical studies of alpha helices suggest that a statistically significant signal should be detectable at (*i*, *i *+ 3) and (*i*, *i *+ 4). Black digits indicate values of *n *significant at *α *= 0.05, and red digits indicate values of *n *significant after Bonferroni adjustment for multiple comparisons, *α *= 0.0025. Each graph (A-J) represents performance with a different algorithm. Where applicable, alignments were recoded with the CHARGE_HIS_2 alphabet, which consistently yielded among the best results. When recoding with CHARGE_HIS_2 was not applicable, LnLCorr and CoMap, the DEF99 and ORIG data sets (respectively) are presented.

The CHARGE_HIS_2 alphabet, where residues are recoded to charged or uncharged with histidine counted as charged, was consistently among the best alphabets for detecting coevolution at (*i*, *i *+ 3) and (*i*, *i *+ 4) in myoglobin. Figure 7 reports p-values for ten algorithms for detecting coevolution at (*i*, *i *+ *n*), for *n *of 1 through 20, where alignments were recoded in the CHARGE_HIS_2 alphabet. (In cases where this specific alphabet recoding was not applicable – LnLCorr and CoMap – results represent the DEF99 and ORIG data sets, respectively.) These graphs are a useful visualization of the relative performance of different algorithms or parameters because positive and negative controls are built-in. Since myoglobin is composed of non-contiguous alpha helices, a signal should be visible at *n *= 3 (*i*, *i *+ 3) and *n *= 4 (*i*, *i *+ 4), and not at other values of *n*. We see that most of the methods identify at least one of these with statistical significance, even after Bonferroni correction for multiple comparisons. AS appears to yield a false positive at *n *= 19.

**Figure 8:**
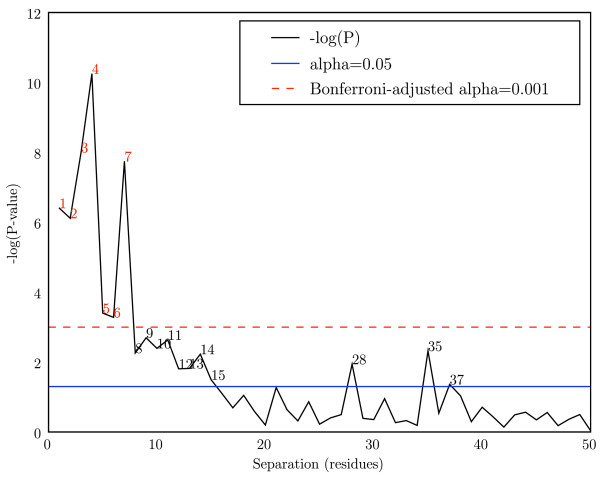
**Mutual Information coevolutionary signal in the myosin rod**. -log(p-values) are presented for each separation of *n *residues (*i*, *i *+ *n*) for *n *ranging from 1 to 50. Biochemical studies of alpha helices suggest that a statistically significant signal should be detectable at (*i*, *i *+ 3) and (*i*, *i *+ 4). Black digits indicate values of *n *significant at *α *= 0.05, and red digits indicate values of *n *significant after Bonferroni adjustment for multiple comparisons, *α *= 0.001. No alphabet recoding was applied before analysis with Mutual Information. In addition to *n *= 3 and *n *= 4, multiples of seven (7,14,21,28,35) have suggestive p-values through *n *= 35.

Figure 8 reports p-values for Mutual Information at separations of 1 through 50 ((*i*, *i *+ 1) through (*i*, *i *+ 50)) in the myosin rod with no alphabet recoding. Since the myosin rod is a contiguous alpha helix and the input alignment contains more data than the myoglobin alignment (in terms of sequence length and number of sequences), some weaker interactions become apparent. While (*i*, *i *+ 3) and (*i*, *i *+ 4) still generate the most significant p-values, other pairs nearby in sequence also show significant p-values. Notably, multiples of seven between seven and thirty-five obtain p-values suggestive of statistical significance, likely resulting from longer-distance stacking interactions in the myosin rod. Alpha helices allow for a comparison of the power of each method not only to detect the strongly coevolving pair sets, but possibly sets of pairs undergoing weaker coevolution as well.

### Long-distance signal in Myosin rod

Figure 8 illustrates that coevolutionary signal is periodically detectable at multiples of seven to distances of thirty-five residues in the myosin rod. Two possible explanations for this signal are: (i) that direct interactions cause coevolution between the residue pairs, or (ii) that indirect interactions cause coevolution between the residue pairs (e.g., residues 1 and 7 directly interact and coevolve, as do residues 7 and 14, and an indirect correlation results in a weaker signal between residues 1 and 14). The data support the latter explanation. The long distance interactions detected in myosin should not be expected in myoglobin, because unlike myosin, the alpha helices in myoglobin are not contiguous.

The period of the alpha helix is 3.6. Direct interactions due to stacking of more distant pairs should therefore occur at multiples of 7.2 (rounded to 7, 14, 22, 29, 36, ...). Instead, we see distant interactions at multiples of 7.0 (separations of 7, 14, 21, 28, and 35 residues). This suggests that the direct interactions occur locally: we see a signal at 35, not at 36, because it is the result of a series of direct local interactions (effectively) 7 positions apart.

The myosin rod is composed of 28 residue homologous units, each composed of four heptad repeats. These repeat regions could be an alternative explanation for the signal at multiples of seven, but this seems less likely. Following evolution of the myosin rod, it is not clear what would drive coevolution of these units, and because conservation reduces MI for a pair of positions, the common ancestry followed by conservation would have the opposite effect.

The process of coevolution is not well understood and there has been very little conclusive evidence about the forces driving coevolution in proteins. In the case of alpha helices, it appears that distant coevolutionary relationships are the result of indirect correlations rather than residue stacking.

## Conclusion

The analyses presented here support our conjecture that robust estimation of coevolution hinges on comparison to a null distribution with equivalent shared ancestry, and that this property can be achieved without explicitly including the tree in the coevolution metric. We demonstrated that transformed tree-ignorant methods detect coevolution with equivalent or better power than tree-aware methods when applied to detect the periodicity of protein alpha helices. A useful next step would involve confirming these results with other evaluations, perhaps based on identifying positions proximal in tertiary structure or individual pairs of coevolving positions which have been biochemically shown to coevolve. The robust statistical properties of transformed tree-ignorant metrics coupled with their generally many orders of magnitude faster computational speed opens up new prospects for detection of coevolving residues within and between proteins. We expect that the application of these methods on a massive scale will provide new insights into the evolution of the structures, functions, and interactions of a wide range of protein families.

## Methods

### Coevolution Detection Methods: tree-ignorant

#### Mutual Information

Mutual information (MI) was calculated over all pairs of positions (columns) in the alignment, as described in [21]. MI is a measure of the degree to which knowing the value of one discrete random variable (in this case, the identity of the amino acid residue at a specific position in a protein) informs you of the value of another discrete random variable (the identity of the residue at another position). Pairs of positions with high MI scores are those that have undergone correlated substitution events.

#### Normalized Mutual Information

Normalized mutual information (NMI) was calculated by dividing the MI score for each pair of alignment positions by the joint entropy of that pair of positions. This normalization, as described in [21], attempts to reduce the minimizing effect of higher sequence conservation on mutual information. For example, if two pairs of columns have perfectly correlated substitutions, but one pair is more highly conserved than the other, the more highly conserved pair will have a lower MI. Normalizing by joint entropy combats this: the pairs will have equal NMI. This method was reimplemented based on the description in [21].

#### Resampled Mutual Information

Resampled mutual information (RMI), introduced here for the first time, is similar to the Jackknife technique but avoids the complication arising from deletion of observations. We use the same resampling approach as reported previously for a tree-dependent probabilistic approach [27] but apply it to the generation of a null distribution of MI for data with arbitrary alphabet sizes. We note here that when the branch lengths on a phylogeny are infinitely long, for a sample of *n *sequences the statistic from the approach of [27] is equivalent to *n *times MI (Easton and Huttley, unpublished). The resampling approach rescales MI by generating permutations of the data for the pair of aligned columns. The modified data sets are identical to the observed data aside for a specific residue whose observed state is replaced by one of the alternate states present in other sequences at that position. As a result, the modified data set has near identical shared ancestry. The frequency with which such permuted data sets result in a MI less than that from the observed data is taken as the probability of observing a permutation with less dependence. Thus, RMI explicitly adjusts for shared ancestry and computes probabilities of coevolution between residue pairs.

#### Statistical Coupling Analysis

Statistical coupling analysis (SCA) was calculated as described in [12], and measures the change in distribution of residues at one position associated with a change in the distribution of residues at another position. If a correlated change in the distribution of residues exists between a pair of positions, that pair is said to be statistically coupled and potentially coevolving.

In addition to the input alignment, SCA requires that the percentage of sequences containing a fixed residue at a position of interest be specified by the user (the cutoff parameter). To study the effect of the cutoff parameter on SCA's performance, we evaluated SCA using six cutoff values: 0.4, 0.5, 0.6, 0.7, 0.8, and 0.9. We reimplemented SCA based on the description of the algorithm in [12], and verified the implementation using the Ranganathan group's Matlab implementation and their published results.

#### Corrected Mutual Information

Corrected mutual information (MIp) was calculated as described in [22]. In attempt to control for 'background MI', or MI arising from random variation or shared ancestry, MI scores are corrected by subtracting the product of the mean MIs for the two positions divided by the overall mean MI score. MIp data were calculated with a Perl implementation of the algorithm provided by the authors.

### Coevolution detection methods: tree-aware

#### Ancestral States

An ancestral-state-reconstruction-based method (AS) for detecting coevolving positions was implemented based on the method described in [4,17]. In this method, ancestral states are inferred for each internal node of the provided phylogenetic tree using maximum likelihood with a substitution model calculated from the alignment and tree. For each pair of positions in the alignment, all pairs of organisms were evaluated to score position pairs on the number of times both underwent a substitution since their last common ancestor (LCA). Scores were calculated as a weighted count of correlated substitutions, by summing the inverse branch lengths between organisms when both residues had changed since the LCA. If neither or only one residue changed since the LCA, nothing was added to the score. This weighting has the effect of favoring correlated changes that happen closer to each other in evolutionary time, since these are less likely to be the results of random substitutions. (Many alternative scoring methods are possible, and a comparison of the various approaches is deferred to a future study.)

Coevolving pairs are expected to score higher than non-coevolving pairs. [17] illustrated that the method for inferring ancestral sequences (parsimony or likelihood, in their analysis), and the method for inferring the phylogenetic tree, have little effect on the ability of this method to detect coevolution.

#### LnLCorr

LnLCorr is available in two versions from the authors. The first (LnLCorr99) [5], is available as a C binary. The second (LnLCorr07) [7], is available as an open source MPI/C++ package. We obtained different results with the different implementations, and therefore present data on both. (Since the methods are very similar, we treat them as a single tree-aware method.) LnLCorr uses a likelihood ratio (LR) to compare the probability of the data under independent and dependent models of evolution. In this implementation, a larger LR between a pair of positions suggests coevolution. (When we refer to LnLCorr, we are discussing properties of the algorithm common to the LnLCorr99 and LnLCorr07 implementations.)

LnLCorr differs from the other methods presented in that it incorporates its own amino acid alphabet reduction step based on a residue metric provided by the user. Each implementation is packaged with a default metric which we refer to as DEF99 and DEF07. At each sequence position, the mean value of the metric is determined, and residues are categorized as above or below the mean. We evaluated LnLCorr using the default metric in addition to the five Atchley metrics (discussed below), but point out that LnLCorr always recodes to a two-state alphabet. These alphabets, while based on the same residue metrics as the alphabets provided to our other methods, result in different encodings of the alignments for LnLCorr than for the other algorithms.

#### Generalized continuous-time Markov process coevolutionary algorithm

The generalized continuous-time Markov process coevolutionary algorithm (GCTMPCA) [3,6] employs maximum likelihood to determine if pairs of substitution events are more likely under a dependent or independent model of evolution. These data were calculated using the C++ implementation provided by the authors as open-source software.

GCTMPCA requires a single parameter *epsilon *(*ε*), the penalty incurred for a single residue change (as opposed to a correlated change between two residues), be provided by the user. Based on empirical evidence the authors define 0.7 as the optimal value of *ε*. Unless otherwise noted we use this default value, which we validated to be optimal from a range of *ε *values. The instantaneous rate matrix for single substitution events must also be provided by the user. The authors use a rate matrix derived from the Dayhoff model [43], and we adopt that as the default. In our studies using reduced amino acid alphabets, this matrix was modified to represent substitution rates in the reduced alphabet. To reduce the substitution matrix, we collapsed the counts and frequencies from the original Dayhoff data in accordance with the reduced amino acid alphabet, and recalculated the instantaneous rate matrix.

#### CoMap

The CoMap algorithm is similar to AS in that it relies on reconstruction of the ancestral states of all positions in the alignment. However, instead of simply counting the number of co-occurring substitutions, CoMap builds 'substitution vectors' for each position, where each element in the vector represents a change in a corresponding branch of the phylogenetic tree. Coevolving positions are identified as those with correlated substitution vectors.

Two variants of CoMap were used in this study. The algorithm presented in [24] builds binary substitution vectors indicating whether a substitution occurred on each branch. In [20] an updated algorithm is presented which incorporates 'weighted substitution vectors', which score changes based on the difference in a pre-specified physicochemical property. Data computed with the binary substitution vector is presented as using an unreduced alphabet (ORIG) since there is no adjustment made for the physicochemical properties of the residues, and data computed using the weighted vectors are presented as reduced-state alphabets since they are designed to represent physicochemical properties of the residues. Three of CoMap's built-in weighting schemes are evaluated (GRANTHAM.POLARITY, GRANTHAM.VOLUME, and CHARGE), in addition to weighting schemes based on the five Atchley factors. CoMap data were calculated with CoMap-1.3.0, provided by the authors.

### Alignments and Trees

#### Myoglobin and Randomized Myoglobin

The first two alignments analyzed were a 153-position myoglobin alignment containing sequences from 42 tetrapods, and a randomized version of this alignment which was used as a negative control. Myoglobin is a mostly alpha helical protein, and therefore serves as a good test case for detecting alpha helix periodicity. This alignment and the corresponding phylogenetic tree were published with [5], where the details of its construction can be found. This alignment contains one gap in one sequence.

The randomized myoglobin alignment was generated by reordering the columns, thereby removing all structural information. Because the periodicity of the alpha helix should no longer be detectable, this alignment served as a negative control. The randomized alignment used the same tree as the myoglobin alignment.

For the purpose of alphabet recoding, it was necessary to replace ambiguous characters in the alignments with non-ambiguous characters. The five B characters and two Z characters in the myoglobin alignment were replaced with D and E, respectively. D and E were chosen over N and Q based on residue frequency in the full alignment. No X characters were present in the myoglobin alignment.

#### Myosin Rod Domain and Randomized Myosin Rod Domain

The myosin heavy chain alignment was originally constructed for the MyoMapr database [44] with ClustalW [14], and subsequently adjusted using PyCogent [45]. Sequences that introduced gaps in other sequences were deleted, as were sequences that contained gaps that were not shared by other sequences at the 99.5% gap identity level (i.e. pairs of sequences left in the alignment were 99.5% identical in gap pattern over the aligned region). A subalignment consisting of only the rod domain was used as the second alpha helical data set.

The rod domain of myosin is a continuous alpha helix which forms a coiled-coil homodimer. This alignment contains 1064 positions, is derived from 114 chordate sequences, and contains no gaps or ambiguous characters. The positions in this alignment were also shuffled to serve as a negative control. A phylogenetic tree was constructed by neighbor-joining [46]. Since the alignment represents a continuous alpha helix, it is ideal for detecting alpha helix periodicity.

### Amino acid alphabets

#### Atchley-factor alphabets

Atchley et al. (2005) calculated values for the twenty canonical amino acid residues by reducing 54 amino acid attributes to five condensed metrics using factor analysis. We used these five metrics (A1 – A5) to define reduced-state amino acid alphabets of varied sizes. To define each 'Atchley-factor alphabet,' we ordered the amino acid residues based on their values for each metric, and then grouped neighboring residues into *n *roughly equal sized groups, where *n *ranged from 2 through 10. Each of the five Atchley factors was used to define nine Atchley-factor alphabets, resulting in forty-five reduced alphabets. We refer to each of these alphabets based on the factor they are derived from and the alphabet size. For example, A1_4 refers to the four-state alphabet derived from Atchley-factor 1 (A1).

The five factors are described in [42] as follows: A1, related to residue polarity; A2, related to propensity for different secondary structures; A3, a molecular size/volume factor; A4, related to amino acid composition in proteins and number of codons; and A5, a electrostatic charge factor.

#### Canonical reduced alphabets

We broke the full twenty-state amino acid alphabet into seven additional reduced-state alphabets based on four commonly recognized features of amino acids: hydropathy index (a three-state alphabet), charge with histidine treated as a charged residue (two- and three-state alphabets), charge with histidine treated as an uncharged residue (two- and three-state alphabets), polarity (a four-state alphabet), and size (a two-state alphabet). These reduced alphabets were generated by splitting the full alphabet based on natural breaks in the properties of the amino acids, and we therefore refer to these as 'rationally-designed' alphabets (as opposed to the heuristically defined Atchley-factor alphabets).

All amino acid alphabet definitions are presented in Table 5.

**Table 5:** Reduced-state alphabet definitions.

(A) Rationally defined alphabets			
Alphabet Identifier	States		
CHARGE_2	KRDE;ACFGHILMNPQSTVWY		

CHARGE_HIS_2	KRDEH;ACFGILMNPPQSTVWY		

CHARGE_3	KR;DE;ACFGHILMNPQSTVWY		

CHARGE_HIS_3	KRH;DE;ACFGILMNPQSTVWY		

SIZE_2	GAVLISPTCND;MFYWQKHRE		

POLARITY_HIS_4	DE;RHK;AILMFPWV;GSTCYNQ		

HYDROPATHY_3	RKDENQH;YWSTG;PAMCFLVI		

(B) Heuristically defined 'Atchley-factor' alphabets			
Alphabet Identifier	States	Alphabet Identifier	States

A1_2	CVILFMWAGS;TPYHQNDERK	A1_3	CVILFMW;AGSTPY;HQNDERK

A1_4	CVILF;MWAGS;TPYHQ;NDERK	A1_5	CVIL;FMWA;GSTP;YHQN;DERK

A1_6	CVI;LFMW;AGS;TPY;HQND;ERK	A1_7	CVI;LFM;WAG;ST;PYH;QND;ERK

A1_8	CVI;LF;MWA;GS;TPY;HQ;NDE;RK	A1_9	CV;IL;FMW;AG;ST;PY;HQN;DE;RK

A1_10	CV;IL;FM;WA;GS;TP;YH;QN;DE;RK		

A2_2	MEALFKIHVQ;RWDTCNYSGP	A2_3	MEALFKI;HVQRWD;TCNYSGP

A2_4	MEALF;KIHVQ;RWDTC;NYSGP	A2_5	MEAL;FKIH;VQRW;DTCN;YSGP

A2_6	MEA;LFKI;HVQ;RWD;TCNY;SGP	A2_7	MEA;LFK;IHV;QR;WDT;CNY;SGP

A2_8	MEA;LF;KIH;VQ;RWD;TC;NYS;GP	A2_9	ME;AL;FKI;HV;QR;WD;TCN;YS;GP

A2_10	ME;AL;FK;IH;VQ;RW;DT;CN;YS;GP		

A3_2	SDQHPLCAVK;WNGERFITMY	A3_3	SDQHPLC;AVKWNG;ERFITMY

A3_4	SDQHP;LCAVK;WNGER;FITMY	A3_5	SDQH;PLCA;VKWN;GERF;ITMY

A3_6	SDQ;HPLC;AVK;WNG;ERFI;TMY	A3_7	SDQ;HPL;CAV;KW;NGE;RFI;TMY

A3_8	SDQ;HP;LCA;VK;WNG;ER;FIT;MY	A3_9	SD;QH;PLC;AV;KW;NG;ERF;IT;MY

A3_10	SD;QH;PL;CA;VK;WN;GE;RF;IT;MY		

A4_2	WHCMYQFKDN;EIPRSTGVLA	A4_3	WHCMYQF;KDNEIP;RSTGVLA

A4_4	WHCMY;QFKDN;EIPRS;TGVLA	A4_5	WHCM;YQFK;DNEI;PRST;GVLA

A4_6	WHC;MYQF;KDN;EIP;RSTG;VLA	A4_7	WHC;MYQ;FKD;NE;IPR;STG;VLA

A4_8	WHC;MY;QFK;DN;EIP;RS;TGV;LA	A4_9	WH;CM;YQF;KD;NE;IP;RST;GV;LA

A4_10	WH;CM;YQ;FK;DN;EI;PR;ST;GV;LA		

A5_2	DSQPVLECWA;HFINMTYKGR	A5_3	DSQPVLE;CWAHFI;NMTYKGR

A5_4	DSQPV;LECWA;HFINM;TYKGR	A5_5	DSQP;VLEC;WAHF;INMT;YKGR

A5_6	DSQ;PVLE;CWA;HFI;NMTY;KGR	A5_7	DSQ;PVL;ECW;AH;FIN;MTY;KGR

A5_8	DSQ;PV;LEC;WA;HFI;NM;TYK;GR	A5_9	DS;QP;VLE;CW;AH;FI;NMT;YK;GR

A5_10	DS;QP;VL;EC;WA;HF;IN;MT;YK;GR		

The 52 reduced-state amino acid alphabets. Each state is defined as a group of characters followed by a semi-colon, so for example, 'KRDEH' and 'ACFGILMNPQSTVWY' are reduced to the charged and uncharged states, respectively, in the CHARGE_HIS_2 alphabet.

#### Correlations between alphabet size and performance

Kendall's *tau *(*τ*) rank correlation test was applied to compute correlation coefficients (*τ*) and p-values for the relationship between alphabet size and method performance. For each Atchley-factor alphabet, the performance at each alphabet size (2 states through 10 states, and the full twenty-state alphabet) were ranked, and the ranks compared against the rank alphabet size to determine if there was a correlation between alphabet size and performance. Performance ranking for a method/alphabet combination was performed based on the -log(p-value) for identifying (*i*, *i *+ 4) pairs as different from the background signal. The (*i*, *i *+ 4) pairs were chosen over the (*i*, *i *+ 3) pairs to define significance because these are generally where the strongest signals were observed for either method, and the biochemical data more strongly support these interactions. A positive *τ *indicates that an increase in the number of states is correlated with an increase in performance.

### Detecting periodicity of the alpha helix

To detect coevolutionary signal resulting from the periodicity of the alpha helix, we compiled coevolution scores for pairs of positions differing by *n *in position number, or (*i*, *i *+ *n*) pairs. For each value of *n*, we compared the distribution of scores for the (*i*, *i *+ *n*) pairs to the distribution of all other positions pairs in the alignment (the background distribution) using a one-tailed (*H*_*a*_: *μ*_*other *_<*μ*_*n*_), two-sample t-test. For example, to look for coevolutionary signal between positions separated by three residues (*i*, *i *+ 4), we compared the distribution of coevolution scores at position pairs {(1,5), (2,6), (3,7), (4,8), ...} with the distribution of all other positions pair coevolution scores {(1,2), (1,3), (1,4), (1,6), ..., (2,3), (2,4), (2,5), (2,7), ...}. When sample sizes are large, the two-sample t-test is robust to deviations of the background distribution from normality and differences in the population sample sizes, making it suitable for this application. An important feature of this test is that methods with higher false positive rates do not achieve more significant p-values. This is because the higher false positive rate tends to increase the coevolution scores of all pairs, thus elevating the background distribution and resulting in a less significant p-value [see Additional file 3].

The multiple comparisons problem is significant, but is unavoidable in full-molecule coevolutionary analyses. We present exact p-values from all tests in relation to several baselines, including the Bonferroni-adjusted alpha to control for multiple comparisons. The Bonferroni adjustment circumvents the multiple comparison problem, but is often considered too stringent, greatly decreasing the power of the statistical test.

The distance matrix structure of the result matrices violates the independence clause of the t-test. To determine if this affected the conclusions, p-values were computed empirically using a non-parametric matrix permutation test [47] for several result matrices. (This method is very computationally intensive, so was not applied to all results.) Results suggest that the p-values obtained from the t-tests are sometimes exaggerated, but the ranking of p-values is consistent. The coevolution score distributions are not normal (see Figure 1), which is also potentially problematic for the t-test. To address this, we applied the Wilcoxon rank-sum test (implemented in the R statistical package) to confirm the results of our t-test p-values, for a selection of result matrices and negative controls. The Wilcoxon p-values also suggest that the t-test p-values are sometimes exaggerated, but rank similarly to the t-test p-values. These results support the use of t-test for comparing the performance of algorithms.

### Identifying individual coevolving pairs

To identify individual coevolving pairs, coevolution scores corresponding to each pair of positions were compared to all coevolution scores in the same matrix using one-observation t-tests to generate a p-value matrix indicating the statistical significance of each score. Significance thresholds (*α*) were varied from 1.0 × 10^-2 ^and 1.0 × 10^-7 ^in steps of one order of magnitude, and statistically significant scores corresponding to (*i*, *i *+ 3) and (*i*, *i *+ 4) pairs were counted as true positives (TP). Statistically significant scores corresponding to all other position pairs were counted as false positives (FP). Scores corresponding to (*i*, *i *+ 3) and (*i*, *i *+ 4) pairs which were not statistically significant were counted as false negatives (FN). This allowed for calculation of precision (P) as *TP/*(*TP *+ *FP*), or statistically significant scores arising from the periodicity of the alpha helix, divided by the total number of statistically significant scores (total hits); and calculation of recall (R) as *TP/*(*TP *+ *FN*), or statistically significant scores arising from the periodicity of the alpha helix, divided by the total number of (*i*, *i *+ 3) and (*i*, *i *+ 4) pairs. We note that pairs are expected to coevolve for effects other than the stability of the alpha helix, and our false positive count is therefore contaminated with many scores which should be counted as true positives. Similarly, not all stacked positions in the alpha helix are expected to coevolve, and our false negative count is therefore contaminated with many true negatives. Since the exact set of coevolving positions is not known, it is not possible to adjust these counts accordingly. This issue is common to all methods however, so while the true precisions and recalls are likely significantly higher than those presented here, the relative values should be meaningful comparisons of the methods. While for simplicity we refer to these scores as precision and recall, more accurately *precision *can be described as the proportion of statistically significant coevolution scores associated with pairs of stacked positions in the alpha helix, and *recall *can be described as the proportion of stacked positions observed to coevolve. We additionally summarize these statistics by presenting F-measures, the harmonic mean of precision and recall, calculated as (2 × *P *× *R*)/(*P *+ *R*).

Precision, recall, F-measure, and total hits data are summarized with area under the curve (AUC) scores for each method, alphabet combination. Six variates of *α *were tested, and AUC was calculated for each metric (Figure 4). Each step in *α *contributed an equal length to the area under that segment of the curve (log_10_*α*^2^- log_10_*α*^1^, opposed to *α*^2 ^- *α*^1^). If no statistically significant scores were returned for one value of *α *(i.e., total hits = 0), precision could not be calculated because the denominator was equal to zero. For these values of *α*, precision was set to zero to facilitate the calculation of AUC. Similarly, when precision and recall were both equal to zero, F-measure could not be calculated and was therefore set to zero. This is convenient because, for example, if a method, alphabet combination achieved no statistically significant hits for any value of *α*, precision AUC was equal to zero. All precision, recall, F-measure, total hits, and AUC data are provided [see Additional file 1].

### *χ*^2 ^comparisons of tree-aware and tree-ignorant methods

The tree-aware and tree-ignorant categories were compared using *χ*^2 ^goodness-of-fit tests (Table 1). A 2 × 2 contingency table was compiled by counting the runs (algorithm/alphabet combinations) in each category which identify a set of pairs as statistically significant with *α *= 0.01. We evaluated performance for identifying (*i*, *i *+ 3) pairs and (*i*, *i *+ 4) pairs in myosin and myoglobin. In each case, counts were tallied using the best and worst performing SCA cutoff values. (SCA cutoff values obtaining the most and least significant median p-values at (*i*, *i *+ 3) and (*i*, *i *+ 4) in each alignment were selected as the best and worst SCA sets, respectively. For example, the (*i*, *i *+ 3) myoglobin tree-ignorant counts in row 1 of Table 1A, and the associated negative control, use cutoff = 0.90 because those values achieved the highest median p-value for that specific data point.) Because SCA performance varies widely with the cutoff value (see Figure 6), and optimization is not always practical, it is useful to see how the methods compare when the best and worst cutoff values are used. Tree-aware counts are always tallied using GCTMPCA runs with the recommended and empirically validated optimal value of *ε *= 0.70.

Comparisons of the tree-aware methods (Table 2) and the tree-ignorant methods (Table 3) were performed similarly. For both categories, 4 × 2 contingency tables were compiled by counting the significant and insignificant scores at (*i*, *i *+ 3) and (*i*, *i *+ 4), with *a *= 0.01. LnLCorr07 and GCTMPCA were not included in the comparison on myosin because the computation time was prohibitive. Counts for GCTMPCA on myoglobin were compiled with *ε *= 0.70. All tree-ignorant methods were compared on myoglobin and myosin, and counts were compiled using the empirically determined optimal SCA cutoff values for each data point.

### Availability of algorithms and data

MI, NMI, RMI, SCA, and AS were implemented in Python and are available as part of the open source PyCogent project [45]. A PyCogent application controller, also available in PyCogent, was developed for GCTMPCA. The original source code for SCA, MIp, and CoMap; the LnLCorr99 command line application; and the LnLCorr07 source code and command line application are available upon request from their authors. The alignment and trees used in these analyses are available in fasta and newick format, respectively [see Additional file 4]. Documentation for regenerating all coevolution score matrices from these alignments and trees is provided [see Additional file 5].

## Authors' contributions

JGC developed the alpha-helix-based approach for comparing algorithms, wrote and executed the software for performing the comparisons, and drafted the manuscript. SS implemented the Statistical Coupling Analysis used in these studies, and assisted with drafting the manuscript. BCE developed the Resampled Mutual Information algorithm. LH assisted with experimental design and drafting of the manuscript. GAH implemented the Resampled Mutual Information used in these studies, and assisted with drafting the manuscript. RK assisted with experimental design, supervised development of the alpha-helix-based approach for comparing algorithms, and assisted with drafting the manuscript.

## Supplementary Material

Additional file 1 **Additional Table 1**. Precision, recall, F-measure, total hits, and AUC data for each method, alphabet pair at different significance thresholds. This zip file contains a csv file containing all data, and a text file containing a description of the data. Click here for file

Additional file 2 **Additional Table 2**. Top five amino acid alphabets for each method on myoglobin and myosin rod alignments. Click here for file

Additional file 3 **Additional Table 3**. The effect of increased false positive rate on t-test p-values. Click here for file

Additional file 4 **Alignments and trees**. The alignments and trees used in the comparisons, in fasta and newick formats, respectively. Click here for file

Additional file 5 **Data recreation**. Documentation, scripts, and supporting data to recreate raw coevolution data. Click here for file
