# Supplementary material for: Detecting coevolution without phylogenetic trees? Tree-ignorant metrics of coevolution perform as well as tree-aware metrics
Source: BMC Evol Biol. 2008 Dec 3;8:327. doi: 10.1186/1471-2148-8-327 (PMC2637866; doi:10.1186/1471-2148-8-327)
Supplement: Additional file 2 — Additional Table 2. Top five amino acid alphabets for each method on myoglobin and myosin rod alignments. [file 1471-2148-8-327-S2.pdf]

Additional Table 2: Top five amino acid alphabets for each method on myoglobin and myosin rod alignments.

(A) Tetrapod Myoglobin

Rank	MI	NMI	RMI	SCA8	MIp	GCTMPCA7	AS
1	CHARGE_HIS_2	A1_4	A3_2	CHARGE_HIS_2	CHARGE_2	A4_7	CHARGE_HIS_3
2	CHARGE_2	A1_3	A3_4	CHARGE_HIS_3	CHARGE_HIS_2	A1_10	CHARGE_2
3	CHARGE_HIS_3	A1_7	A4_4	A1_7	CHARGE_3	A4_10	CHARGE_3
4	A1_4	CHARGE_HIS_2	A5_10	A1_4	CHARGE_HIS_3	A5_5	CHARGE_HIS_2
5	CHARGE_3	CHARGE_2	A4_7	A1_2	A4_4	A5_10	POLARITY_HIS_4

(B) Chordate Myosin

Rank	MI	NMI	RMI	SCA6	MIp	LnLCorr99	AS	CoMap
1	CHARGE_3	CHARGE_3	A4_3	CHARGE_3	A5_9	DEF99	A1_2	ORIG
2	CHARGE_2	CHARGE_2	A4_4	CHARGE_HIS_3	A2_9	A4	A5_5	A4
3	CHARGE_HIS_3	CHARGE_HIS_3	A3_3	CHARGE_2	A5_10	A5	CHARGE_3	VOLUME
4	CHARGE_HIS_2	CHARGE_HIS_2	A3_4	CHARGE_HIS_2	ORIG	A2	A2_5	A2
5	A3_9	A4_4	CHARGE_2	SIZE_2	A3_9	A1	A5_9	A1

For each method and alignment combination, the top five ranking alphabets are presented. Rankings are determined based on the p-value at $(i, i+4)$ in the given alignment. Methods that did not achieve any significant p-values (at $\alpha = 0.01$) for the given alignment are not included as those rankings are not meaningful.
