# Supplementary material for: Detecting coevolution without phylogenetic trees? Tree-ignorant metrics of coevolution perform as well as tree-aware metrics
Source: BMC Evol Biol. 2008 Dec 3;8:327. doi: 10.1186/1471-2148-8-327 (PMC2637866; doi:10.1186/1471-2148-8-327)
Supplement: Additional file 3 — Additional Table 3. The effect of increased false positive rate on t-test p-values. [file 1471-2148-8-327-S3.pdf]

Additional Table 3: The effect of increased false positive rate on t-test p-values.

Alignment, Alphabet	p-value with (m,n) additional 1.0 scores in the *(i,i+4)* and background distributions, respectively					
	(0,0)	(1,82)	(10,820)	(20,1640)	(50,4100)	(100,8200)
Tetrapod Myoglobin, `charge_his_2`	5.6×10^{-5}	0.5×10^{-2}	2.6×10^{-1}	3.0×10^{-1}	3.5×10^{-1}	3.6×10^{-1}
	(0,0)	(1,539)	(10,5390)	(20,10780)	(50,26950)	(100,53900)
Chordate Myosin, `orig`	1.67×10^{-6}	3.35×10^{-6}	1.47×10^{-4}	1.05×10^{-3}	1.0×10^{-2}	3.87×10^{-2}

To illustrate that methods with increased false positive rates achieve less significant p-values in the t-test-based analysis, for top performing method/alphabet combinations $(i, i{+}4)$ score distributions are compared against background distributions, where both distributions have been modified to include additional high (i.e., positive) scores. False positives are added to both the coevolving and non-coevolving distributions, at the same rate. For example, when 1 false positive is added to the myoglobin $(i, i+4)$, 82 false positives are added to the background distribution because the background distribution contains 82 times more values than the $(i, i+4)$ distribution. The additional high scores are analogous to extreme false positives (since the max value of 1.0 for NMI was added).
